# Supplementary material for: Association of Computed Tomography Scan-Assessed Body Composition with Immune and PI3K/AKT Pathway Proteins in Distinct Breast Cancer Tumor Components
Source: Int J Mol Sci. 2024 Dec 14;25(24):13428. doi: 10.3390/ijms252413428 (PMC11676426; doi:10.3390/ijms252413428)
Supplement: Supplementary file 1 [file ijms-25-13428-s001.zip › ijms-3335588-supplementary.pdf]

Supplementary Materials

Supplemental Table S1. Areas of specific body composition components (cm^2) (N = 52)

Body composition component	Mean	SD	Median	Interquartile range
VAT	124.4	80.6	124.2	61.0 – 172.4
SAT	304.6	136.6	303.8	222.9 – 378.2
IMAT	12.5	23.5	7.9	4.9 – 11.7
VLDM	17.6	9.7	15.7	11.2 – 23.0
LDM	39.9	15.3	40.6	28.9 – 47.0
NDM	61.8	23.3	64.7	47.3 – 78.5
HDM	3.7	1.8	3.3	2.2 – 5.1
VHDM	3.4	1.3	3.4	2.8 – 4.1
TAT	441.6	188.3	453.4	316.2 – 552.2
TSM	126.5	20.6	124.0	113.5 – 137.9

Abbreviations: VAT, visceral adipose tissue; SAT, subcutaneous adipose tissue; IMAT, intermuscular adipose tissue; VLDM, very low-density muscle; LDM, low-density muscle; NDM, normal-density muscle; HDM, high-density muscle; VHDM, very high-density muscle; TAT, total adipose tissue; TSM, total skeleton muscle tissue.

Supplemental Table S2. Body composition group by patient and clinical characteristics

Characteristic	Body composition type (N = 52)			
	Adequate	High adiposity	Low muscle	Low muscle/high adiposity
N (% total)	22 (42.3%)	13 (25.0%)	14 (26.9%)	3 (5.8%)
Age, mean (SD), years	54.5 (10.9)	59.1 (9.1)	58.3 (11.4)	57.7 (10.2)
Age category, %				
<55	12 (54.6%)	4 (30.8%)	6 (42.9%)	1 (33.3%)
≥55	10 (45.4%)	9 (69.2%)	8 (57.1%)	2 (66.7%)
Race, N (col %)				
White	17 (77.3%)	11 (84.6%)	12 (85.7%)	3 (100.0%)
Black	5 (22.7%)	2 (15.4%)	2 (14.3%)	0 (0.0%)
AJCC stage, N (col %)				
I	7 (31.8%)	4 (30.8%)	5 (35.7%)	1 (33.3%)
II	11 (50.0%)	8 (61.5%)	6 (42.9%)	1 (33.3%)
III	4 (18.2%)	1 (7.7%)	3 (21.4%)	1 (33.3%)
Tumor grade, N (col %)				
I	1 (4.6%)	4 (30.8%)	0 (0.0%)	0 (0.0%)
II	11 (50.0%)	6 (46.1%)	8 (57.1%)	3 (100.0%)
III	10 (45.4%)	3 (23.1%)	6 (42.9%)	0 (0.0%)
Tumor size, N (col %)				
<25 mm	8 (36.4%)	8 (61.5%	7 (50.0%)	1 (33.3%)
≥25 mm	9 (40.9%)	4 (30.8%)	4 (28.6%)	0 (0.0%)
Missing	5 (22.7%)	1 (7.7%)	3 (21.4%)	2 (66.7%)
ER status, N (col %)				
Positive	18 (81.8%)	13 (100.0%)	12 (85.7%)	3 (100.0%)
Negative	4 (18.2%)	0 (0.0%)	2 (14.3%)	0 (0.0%)
PR status, N (col %)				
Positive	15 (68.2%)	13 (100.0%)	12 (85.7%)	3 (100.0%)
Negative	7 (31.8%)	0 (0.0%)	2 (14.3%)	0 (0.0%)
HER2 status, N (col %)				
Positive	7 (31.8%)	0 (0.0%)	3 (21.4%)	0 (0.0%)
Negative	15 (68.2%)	13 (100.0%)	11 (78.6%)	2 (66.7%)
Missing	0 (0.0%)	0 (0.0%)	0 (0.0%)	1 (33.3%)

Abbreviations: AJCC, American Joint Committee on Cancer; ER; ER, estrogen receptor; PR, progesterone receptor

Supplemental Table S3. Percentages of obesity and sarcopenia in each body composition type

Body composition type	Overall[a]	Adequate	High adiposity	Low muscle	High adiposity with low muscle
N (% total)	43 (100%)	18 (41.9%)	11 (25.5%)	11 (25.5%)	3 (7.1%)
Obesity					
No (BMI <30 kg/m^2)	23 (53.5%)	10 (55.6%)	2 (18.2%)	9 (81.8%)	2 (66.7%)
Yes (BMI ≥30 kg/m^2)	20 (46.5%)	8 (44.4%)	9 (81.8%)	2 (18.2%)	1 (33.3%)
Sarcopenia[b]					
No	38 (88.4%)	17 (94.4%)	11 (100%)	8 (72.7%)	2 (66.7%)
Yes	5 (11.6%)	1 (5.6%)	0 (0%)	3 (27.3%)	1 (33.3%)

[a] 9 patients with missing height or weight for body mass index (BMI) calculation were not included.
[b] Sarcopenia was defined as skeletal muscle index (SMI) < 38.9 cm^2/m^2. SMI was defined as total skeletal muscle area at L3 in cm^2 divided by height in meters squared.

Supplemental Table S4. Hazard ratios (HRs) of mortality, by body composition type

Model	Body composition type (N = 168, 25 deaths)			
	Adequate	High adiposity	Low muscle	Low muscle/high adiposity
No. in each group	67	47	44	10
Deaths, No.	8	7	8	2
Model 1, HR (95% CI)	1.00 (reference)	1.19 (0.43-3.29)	1.51 (0.57-4.04)	1.52 (0.32-7.16)
Model 2, HR (95% CI)	1.00 (reference)	1.03 (0.37-2.90)	2.21 (0.79-6.19)	1.78 (0.37-8.46)

Model 1: Cox proportional hazard model without covariates

Model 2: Cox proportional hazard model adjusting age at diagnosis, race, breast cancer stage, and tumor grade

Tertiles of TAT: 368.0 and 518.9 cm^2; tertiles of TSM: 117.9 and 135.5 cm^2

Supplemental Table S5. Protein marker expression level[a] by clinical characteristic. Limited to the proteins significantly associated with body composition type shown in Table 3.

**Tissue compartment**	Tumor		Stroma					
**Marker**	Pan-AKT	CTLA4	INPP4B	CD8	CD45RO	GZMB	CD20	CD3
**AJCC stage, %**								
I	5.65 (0.37)	0.32 (0.57)	2.84 (0.32)	4.09 (0.44)	1.84 (0.36)	3.12 (0.38)	0.82 (0.24)	2.68 (0.47)
II	5.64 (0.30)	0.41 (0.46)	2.85 (0.26)	3.28 (0.36)	1.56 (0.30)	3.36 (0.31)	0.68 (0.20)	2.26 (0.38)
III	7.16 (0.54)	1.76 (0.82)	3.33 (0.46)	3.48 (0.62)	1.37 (0.51)	3.86 (0.54)	1.62 (0.34)	2.91 (0.66)
**Tumor grade**								
I	5.93 (0.73)	-0.08 (1.05)	3.17 (0.58)	4.15 (0.80)	2.14 (0.64)	4.12 (0.68)	0.80 (0.46)	3.04 (0.84)
II	5,95 (0.31)	0.91 (0.45)	2.87 (0.26)	3.57 (0.36)	1.50 (0.29)	3.36 (0.31)	0.86 (0.21)	2.53 (0.38)
III	5.83 (0.37)	0.33 (0.54)	2.93 (0.31)	3.44 (0.42)	1.61 (0.34)	3.15 (0.36)	0.94 (0.24)	2.34 (0.44)
**Tumor size**								
<25 mm	6.01 (0.34)	0.43 (0.47)	3.09 (0.27)	3.74 (0.38)	1.53 (0.31)	3.34 (0.31)	0.90 (0.21)	2.47 (0.41)
≥25 mm	6.06 (0.39)	1.54 (0.55)	3.19 (0.30)	3.81 (0.43)	1.99 (0.35)	3.97 (0.35)	1.18 (0.24)	2.89 (0.45)
Missing	5.43 (0.49)	-0.51 (0.68)	2.15 (0.39)	2.86 (0.56)	1.15 (0.45)	2.38 (0.45)	0.35 (0.31)	1.94 (0.59)
**ER status**								
Positive	5.90 (0.24)	0.54 (0.35)	2.93 (0.20)	3.68 (0.28)	1.63 (0.22)	3.47 (0.23)	0.90 (0.16)	2.60 (0.29)
Negative	5.91 (0.66)	1.01 (0.97)	2.92 (0.53)	2.87 (0.73)	1.49 (0.59)	2.63 (0.62)	0.79 (0.42)	1.85 (0.77)
**PR status**								
Positive	5.89 (0.25)	0.55 (0.37)	3.01 (0.21)	3.64 (0.29)	1.59 (0.23)	3.45 (0.25)	0.86 (0.16)	2.51 (0.30)
Negative	5.97 (0.54)	0.80 (0.79)	2.54 (0.43)	3.33 (0.60)	1.70 (0.48)	2.99 (0.51)	0.98 (0.34)	2.49 (0.63)
**HER2 status**								
Positive	6.33 (0.51)	0.88 (0.75)	3.25 (0.43)	3.81 (0.60)	1.69 (0.48)	3.80 (0.51)	0.98 (0.34)	3.09 (0.63)
Negative	5.79 (0.25)	0.52 (0.37)	2.85 (0.21)	3.53 (0.29)	1.59 (0.23)	3.26 (0.25)	0.86 (0.16)	2.38 (0.30)

Abbreviations: AJCC, American Joint Committee on Cancer; ER; ER, estrogen receptor; PR, progesterone receptor

[a] Least square means (standard error) estimated from mixed effect models.

Supplemental Table S6. Associations between body composition type and immune and PI3K/AKT protein expression levels in breast tumors, with additional adjustment of chemotherapy receipt

Body composition type (compared with adequate type)	Protein marker	Tissue compartment	Estimate (log2-fold change)[a]	P value
Low muscle	Pan-AKT	Tumor	-0.79	0.042
Low muscle	CTLA4	Tumor	0.54	0.033
Low muscle	INPP4B	Stroma	1.17	<0.0001
High adiposity	INPP4B	Stroma	0.64	0.036
High adiposity	CD8	Stroma	1.32	0.009
High adiposity	CD45RO	Stroma	0.98	0.021
High adiposity	GZMB	Stroma	0.70	0.08
High adiposity	CD20	Stroma	0.59	0.009
High adiposity	CD3	Stroma	1.08	0.056
High adiposity/low muscle	Phospho-tuberin (T1462)	Tumor	-1.42	0.017
High adiposity/low muscle	Phospho-PRAS40 (T246)	Tumor	-1.93	0.024
High adiposity/low muscle	Phospho-PRAS40 (T246)	Stroma	-1.15	0.058
High adiposity/low muscle	CTLA4	Stroma	1.25	0.009
High adiposity/low muscle	PD-1	Stroma	-1.43	0.010
High adiposity/low muscle	CD14	Stroma	-2.04	0.019
High adiposity/low muscle	PLCG1	Stroma	-1.21	0.024

[a] Model adjusted for analytical batch, race, breast cancer stage, tumor grade, and chemotherapy

Supplemental Table S7. Associations of protein markers by tertiles (T) of muscle for the significant associations of the low muscle body composition type

Protein marker (tissue compartment)	Body fatness measurements: exposure vs referent groups	Estimate (log2-fold change)	P value
Pan-AKT (tumor)	TSM T2 vs. T3	0.41	0.44
	TSM T1 vs. T3	-0.51	0.48
	TSM T1 vs. T2+T3	-0.78	0.057
	Low muscle vs. adequate	-0.92	0.043
CTLA4 (tumor)	TSM T2 vs. T3	0.14	0.30
	TSM T1 vs. T3	0.29	0.33
	TSM T1 vs. T2+T3	0.27	0.30
	Low muscle vs. adequate	0.53	0.059
INPP4B (stroma)	TSM T2 vs. T3	0.09	0.77
	TSM T1 vs. T3	1.22	0.0009
	TSM T1 vs. T2+T3	1.01	0.0009
	Low muscle vs. adequate	1.18	0.0003

All models adjust for analytical batch, race, breast cancer stage, and tumor grade. The total skeletal muscle (TSM) models additionally adjust for total adipose tissue tertiles.

Supplemental Table S8. Associations of protein markers for tertiles (T) of adiposity and WC for the significant associations of the high adiposity body composition type

Protein marker (tissue compartment)	Body fatness measurements: exposure vs referent groups	Estimate (log2-fold change)	P value
INPP4B (stroma)	TAT T2 vs. T1	0.42	0.13
	TAT T3 vs. T1	0.86	0.017
	TAT T3 vs. T2+T1	0.43	0.16
	High adiposity vs. adequate	0.64	0.058
	WC T2 vs. T1	0.03	0.94
	WC T3 vs. T1	0.48	0.18
CD8 (stroma)	TAT T2 vs. T1	0.16	0.76
	TAT T3 vs. T1	0.37	0.56
	TAT T3 vs. T2+T1	0.70	0.18
	High adiposity vs. adequate	1.16	0.043
	WC T2 vs. T1	0.06	0.92
	WC T3 vs. T1	0.27	0.62
CD45RO (stroma)	TAT T2 vs T1	0.53	0.21
	TAT T3 vs. T1	0.94	0.07
	TAT T3 vs. T2+T1	0.76	0.07
	High adiposity vs. adequate	0.95	0.043
	WC T2 vs. T1	0.03	0.96
	WC T3 vs. T1	0.30	0.65
GZMB (stroma)	TAT T2 vs. T1	0.10	0.81
	TAT T3 vs. T1	0.28	0.58
	TAT T3 vs. T2+T1	0.15	0.72
	High adiposity vs. adequate	0.63	0.17
	WC T2 vs. T1	0.34	0.48
	WC T3 vs. T1	0.41	0.36
CD20 (stroma)	TAT T2 vs. T1	0.004	0.99
	TAT T3 vs. T1	0.20	0.54
	TAT T3 vs. T2+T1	0.33	0.20
	High adiposity vs. adequate	0.61	0.031
	WC T2 vs. T1	0.23	0.42
	WC T3 vs. T1	0.45	0.09
CD3 (stroma)	TAT T2 vs. T1	0.21	0.72
	TAT T3 vs. T1	0.35	0.62
	TAT T3 vs. T2+T1	0.60	0.29
	High adiposity vs. adequate	1.07	0.09
	WC T2 vs. T1	0.06	0.93
	WC T3 vs. T1	0.10	0.86

All models adjust for analytical batch, race, breast cancer stage, and tumor grade. The total adipose tissue (TAT) and waist circumference (WC) models additionally adjust for total skeletal muscle tertiles.

Supplemental Table S9. Associations of protein markers for body mass index and high adiposity for the markers significantly associated with the high adiposity body composition type, restricted to participants with data on body mass index (N= 40)

Protein marker (tissue compartment)	Body fatness measurement: exposure vs. referent groups	Estimate (log2-fold change)	P value
INPP4B (stroma)	High adiposity vs. adequate	0.63	0.07
	Overweight vs. normal weight	-0.40	0.36
	Obesity vs. normal weight	-0.15	0.76
CD8 (stroma)	High adiposity vs. adequate	1.97	0.001
	Overweight vs. normal weight	-0.47	0.48
	Obesity vs. normal weight	0.29	0.71
CD45RO (stroma)	High adiposity vs. adequate	1.51	0.0025
	Overweight vs. normal weight	0.13	0.81
	Obesity vs. normal weight	0.16	0.80
GZMB (stroma)	High adiposity vs. adequate	0.47	0.28
	Overweight vs. normal weight	-0.76	0.12
	Obesity vs. normal weight	-0.39	0.48
CD20 (stroma)	High adiposity vs. adequate	0.64	0.0057
	Overweight vs. normal weight	-0.49	0.05
	Obesity vs. normal weight	0.06	0.83
CD3 (stroma)	High adiposity vs. adequate	1.68	0.0124
	Overweight vs. normal weight	-0.28	0.70
	Obesity vs. normal weight	0.48	0.57

All models adjust for analytical batch, race, breast cancer stage, and tumor grade.

Supplemental Table S10. Statistical power of protein markers in the tumor and stromal compartments

	Marker	Power	
		Tumor	Stromal
1	CD8	0.170	0.545
2	GZMB	0.125	0.625
3	CD11c	0.120	0.610
4	PD-L1	0.330	0.435
5	CTLA4	0.645	0.615
6	Beta-2-microglobulin	0.340	0.420
7	CD20	0.120	0.640
8	CD68	0.140	0.420
9	SMA	0.100	0.490
10	Fibronectin	0.205	0.385
11	CD45	0.105	0.540
12	PanCk	0.155	0.165
13	HLA-DR	0.370	0.525
14	Ki-67	0.375	0.485
15	PD-1	0.360	0.500
16	CD56	0.230	0.325
17	CD3	0.170	0.475
18	CD4	0.110	0.460
19	CD45RO	0.315	0.450
20	FOXP3	0.265	0.200
21	CD163	0.395	0.530
22	CD66b	0.260	0.235
23	CD14	0.240	0.695
24	FAP-alpha	0.400	0.360
25	CD34	0.215	0.670
26	Pan-AKT	0.500	0.385
27	Phospho-GSK3B (S9)	0.365	0.380
28	PLCG1	0.350	0.590
29	Phospho-Tuberin (T1462)	0.700	0.315
30	Phospho-AKT1 (S473)	0.435	0.465
31	Phospho-GSK3A (S21)/Phospho-GSK3B (S9)	0.360	0.325
32	MET	0.285	0.510
33	Phospho-PRAS40 (T246)	0.565	0.560
34	INPP4B	0.495	0.960
35	PTEN	0.265	0.740
36	PR	0.305	0.510
37	ER-alpha	0.405	0.485
38	EpCAM	0.290	0.210

39	Her2	0.595	0.485
40	MART1	0.190	0.275
41	Bcl-2	0.470	0.525
42	S100B	0.165	0.525
43	NY-ESO-1	0.405	0.490

Supplemental Table S11. Characteristics of patients with tumor samples on tissue microarrays (TMAs) vs. patients without or with ineligible tumor samples vs. patient records from the Tumor Registry at UF Health

Characteristic	Patients with tumor samples on TMA[a]	Patients without or with ineligible tumor samples	Tumor Registry at UF Health[b]
	Mean (SD) or No. (%)	Mean (SD) or No. (%)	Mean (SD) or No. (%)
**N**	57 (100%)	527 (100%)	2223 (100%)
Age at diagnosis (years)	57.1 (10.7)	58.3 (11.2)	58.6 (12.6)
**Race**			
White	48 (84.2%)	412 (79.4%)	1809 (82.3%)
Black	9 (15.8%)	107 (20.6%)	313 (14.3%)
Asian	0 (0%)	0 (0%)	35 (1.6%)
PI/AI	0 (0%)	0 (0%)	2 (0.1%)
Others	0 (0%)	0 (0%)	38 (1.7%)
Missing	0	8	26
**AJCC clinical stage**			
0	0 (0%)	5 (1.2%)	430 (21.7%)
I	21 (36.8%)	167 (41.5%)	901 (45.6%)
II	27 (47.3%)	127 (31.6%)	401 (20.3%)
III	9 (15.8%)	59 (14.7%)	105 (5.3%)
IV	0 (0%)	44 (10.9%)	141 (7.1%)
Missing	0	125	245
**Tumor grade**			NA
I	5 (8.8%)	50 (13.5%)	
II	29 (50.9%)	145 (39.1%)	
III	23 (40.3%)	176 (47.4%)	
Missing	0	156	
**Tumor size**			NA
<25 mm	26 (56.5%)	165 (58.1%)	
≥25 mm	10 (43.5%)	119 (41.9%)	
Missing	11	243	
**ER status**			
Positive	49 (86.0%)	299 (76.3%)	1724 (82.8%)
Negative	8 (14.0%)	93 (23.7%)	357 (17.2%)
Missing	0	135	142
**PR status**			
Positive	46 (80.7%)	264 (68.2%)	1538 (74.5%)
Negative	11 (19.3%)	123 (31.8%)	526 (25.5%)
Missing	0	140	159
**HER2 status**			
Positive/equivocal	10 (17.9%)	68 (20.1%)	284 (19.7%)

Negative	46 (82.1%)	270 (79.9%)	1158 (80.3%)
Missing/test not done	1	189	781
**Year of CT scan**			NA
2011	0 (0%)	16 (3.4%)	
2012	1 (1.9%)	59 (12.4%)	
2013	13 (24.1%)	83 (17.5%)	
2014	8 (14.8%)	59 (12.4%)	
2015	6 (11.1%)	67 (14.1%)	
2016	3 (5.6%)	81 (17.1%)	
2017	9 (16.7%)	61 (12.8%)	
2018	12 (22.2%)	27 (5.7%)	
2019	2 (3.7%)	20 (4.2%)	
2020	0 (0%)	2 (0.4%)	
Missing	3	52	
**Year of diagnosis**			
Before 2011	0 (0%)	98 (20.9%)	519 (23.4%)
2011	3 (5.3%)	35 (7.5%)	210 (9.5%)
2012	3 (5.3%)	41 (8.8%)	210 (9.5%)
2013	12 (21.1%)	41 (8.8%)	235 (10.6%)
2014	4 (7.0%)	53 (11.3%)	240 (10.8%)
2015	7 (12.3%)	50 (10.7%)	263 (11.8%)
2016	4 (7.0%)	53 (11.3%)	257 (11.6%)
2017	8 (14.0%)	39 (8.3%)	277 (12.5%)
2018	10 (17.5%)	32 (6.8%)	12 (0.5%)
2019	6 (10.5%)	15 (3.2%)	NA
After 2019	0 (0%)	11 (2.4%)	NA
Missing	0	59	0

Abbreviations: AJCC, American Joint Committee on Cancer; CT, computed tomography; ER; ER, estrogen receptor; NA, not available; PI/AI, Pacific Islanders/American Indians; PR, progesterone receptor

[a] Including 52 patients who had GeoMx Digital Spatial Profiler complete data and 5 patients who had tumor tissue on tissue microarrays but the tissue failed during the assay.

[b] Restricted to females and analytic cases (class of case 00-22, patients who were diagnosed or administered any of their first course of treatment at the accessioning facility after the registry's reference date are analytic), 2008 to January 2018.

Supplemental Table S12. Time interval (days) between computed tomography (CT) scan procedure and breast cancer diagnosis and between CT scan and surgery, the time when the tumor samples were obtained

	No.	Mean	SD	Median	Interquartile range
**Time between CT and diagnosis**					
CT performed before diagnosis	20	237	233	173	74 – 344
CT performed after diagnosis	32	352	450	111	35 – 592
**Time between CT and surgery[a]**					
CT performed before surgery	25	273	412	121	23 – 253
CT performed after surgery	24	235	244	173	29 – 346

[a] Three patients had missing data on the date of surgery.

Supplemental Table S13. Tissue localization sites and Hounsfield unit cut points used for computed tomography scan annotations

**Tissue**	**Localization sites**	**Hounsfield unit, lower limit**	**Hounsfield unit, upper limit**
Subcutaneous adipose tissue (SAT)	Subcutaneous fat, skin	-190	-30
Intermuscular Adipose tissue (IMAT)	Inter-muscular fat, marrow[a]	-190	-30
Visceral Adipose tissue (VAT)	Fat around organs	-150	-50
Very low-density muscle (VLDM)	Muscle-fat boarders, skin, marrow	-29	0
Low density muscle (LDM)	Inter- & intra-muscular, skin, muscle-fat, and marrow borders	0	35
Normal density muscle (NDM)	Muscle, skin, marrow	35	101
High density muscle (HDM)	Bone-muscle border, intra-muscular	101	151
Very high-density muscle (VHDM)	Bone-muscle and bone-marrow boarders	151	200

[a] Marrow was excluded from annotations.

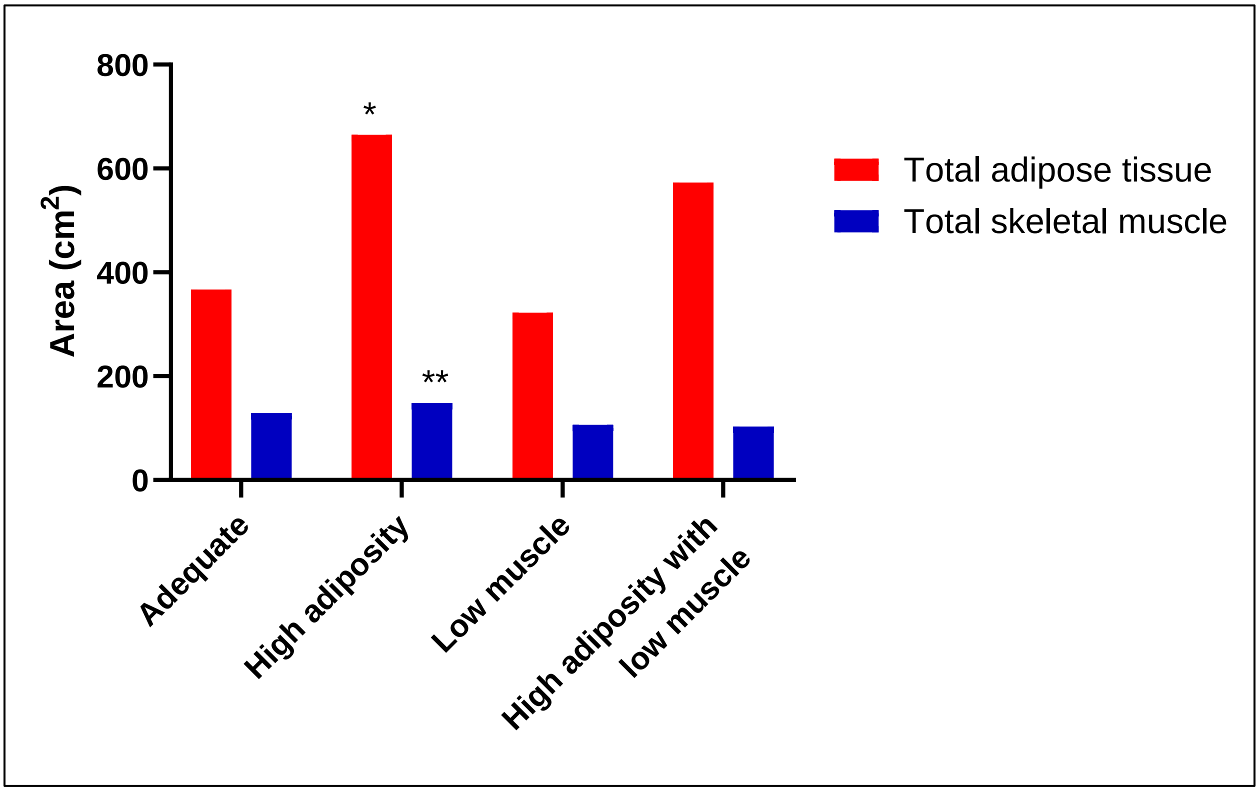

Supplemental Figure S1. Total adipose and muscle tissue areas by body composition phenotype.

* P<0.05 (t-tests) compared with the adequate and the low muscle groups.

** P<0.05 (t-tests) compared with the adequate, the low muscle, and the high adiposity with low muscle groups

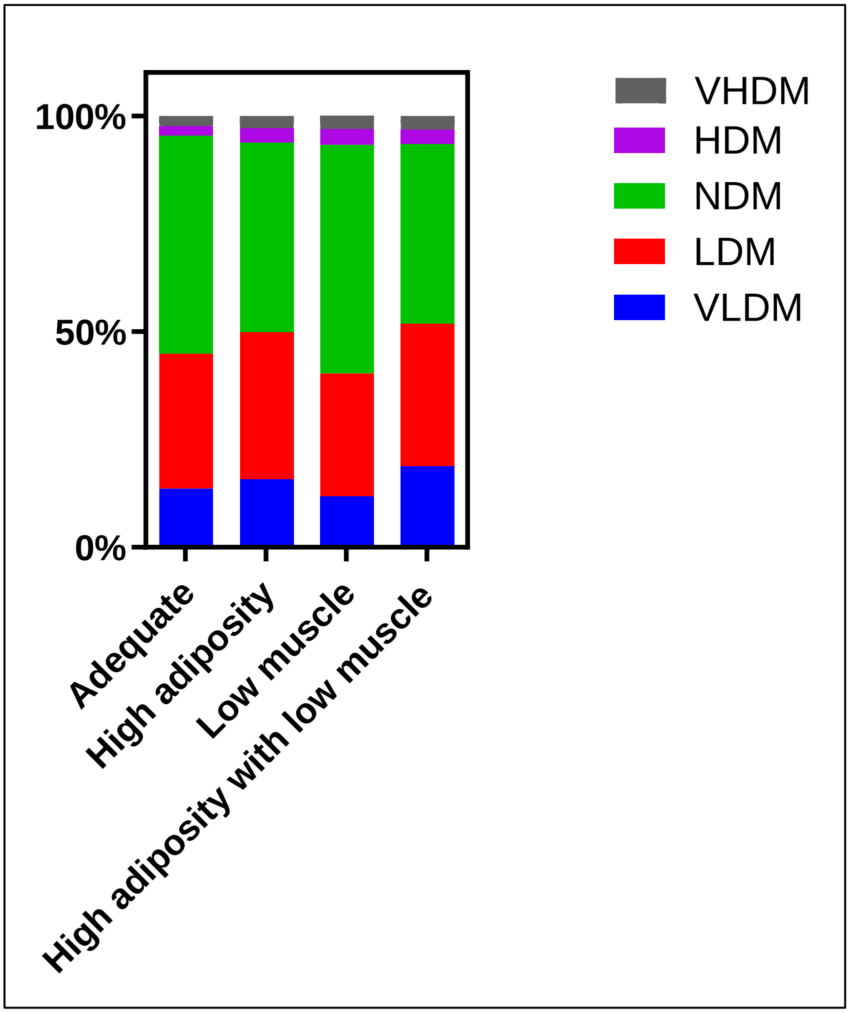

Supplemental Figure S2. Percentages of different densities of muscle in total skeletal muscle area by body composition phenotype. Abbreviations: VHDM, very high-density muscle; HDM, high-density muscle; NDM, normal-density muscle; LDM, low-density muscle; VLDM, very low-density muscle

$P>0.05$ for each muscle type between body composition groups (two proportion Z-tests)

A

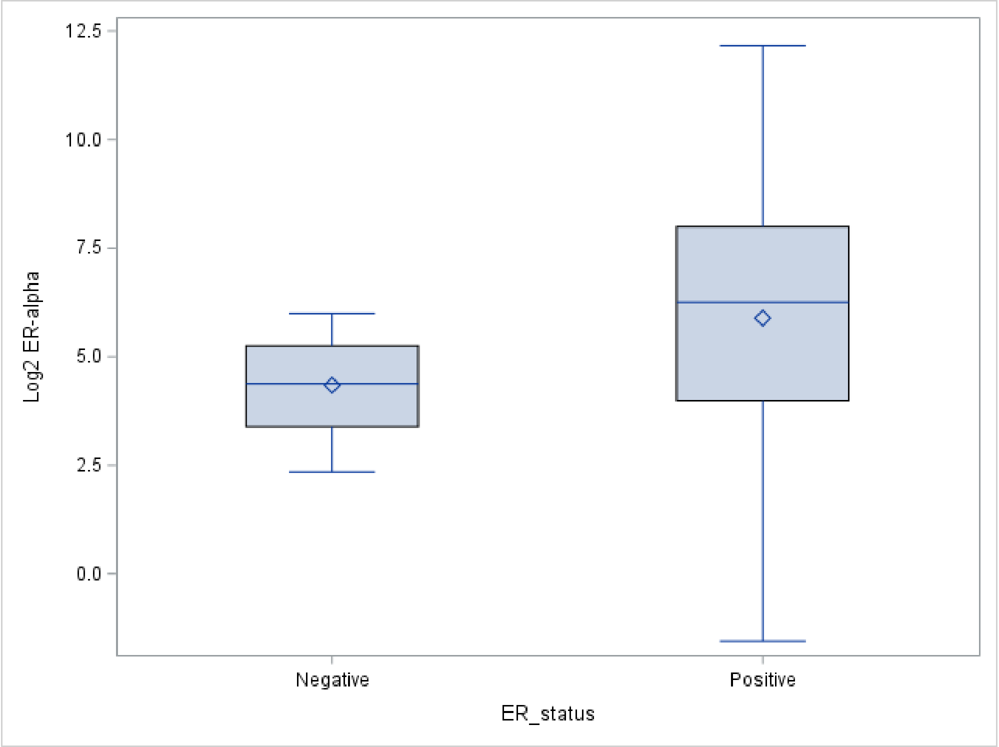

12.5
10.0
7.5
5.0
2.5
0.0
Log2 ER-alpha
Negative
Positive
ER_status

B

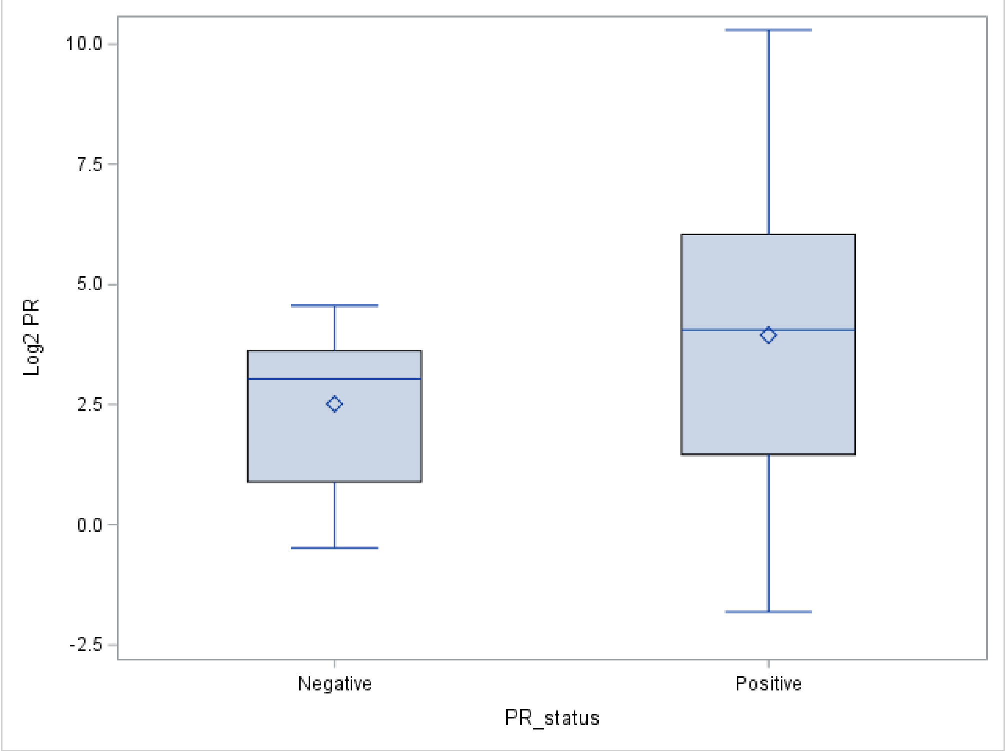

10.0
7.5
5.0
2.5
0.0
-2.5
Log2 PR
Negative
Positive
PR_status

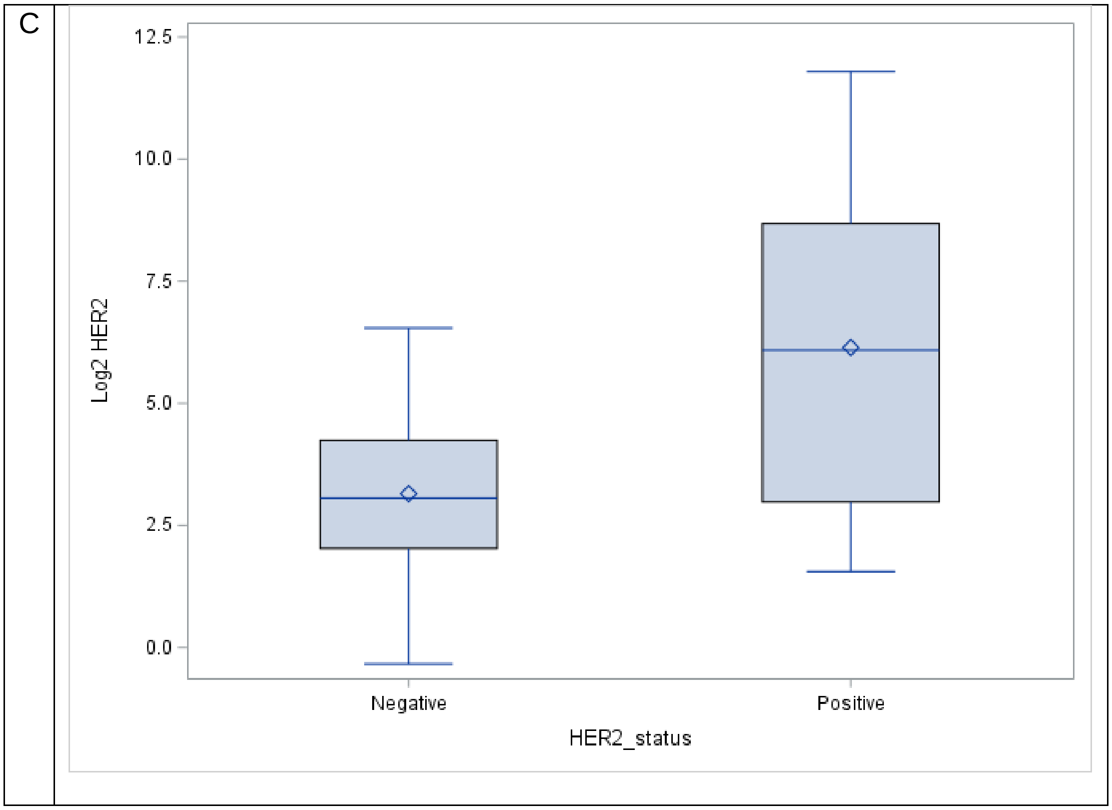

Supplemental Figure S3. Box and whisker plots showing levels of estrogen receptor (ER)-alpha, progesterone receptor (PR), and HER2 proteins in the epithelium compartment measured using a GeoMx Digital Spatial Profiler plotted by ER, PR, and HER2 status obtained from pathology reports (immunohistochemistry). Panel A: ER; Panel B: PR; Panel C: HER2. Horizontal lines indicate medians; outer edges of boxes, Q1 and Q3 values; error bars, minimum and maximum values. Between-group differences were examined using linear mixed models ($P=0.20$ for ER, $P=0.13$ for PR, and $P<0.001$ for HER2).

A

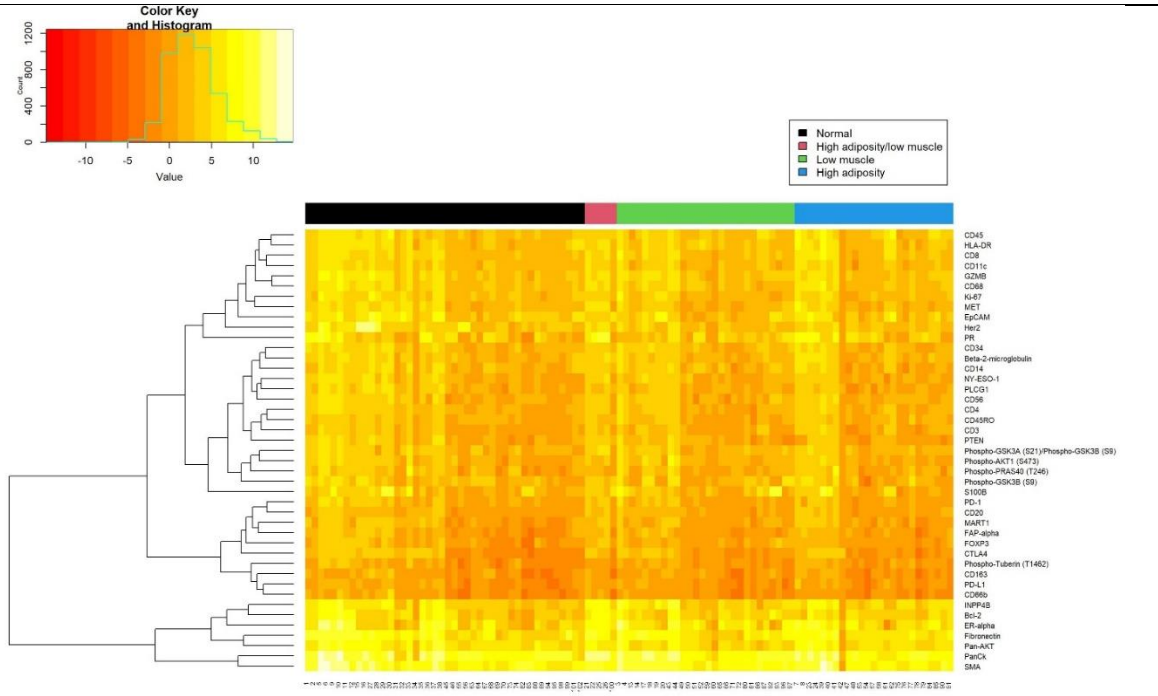

B

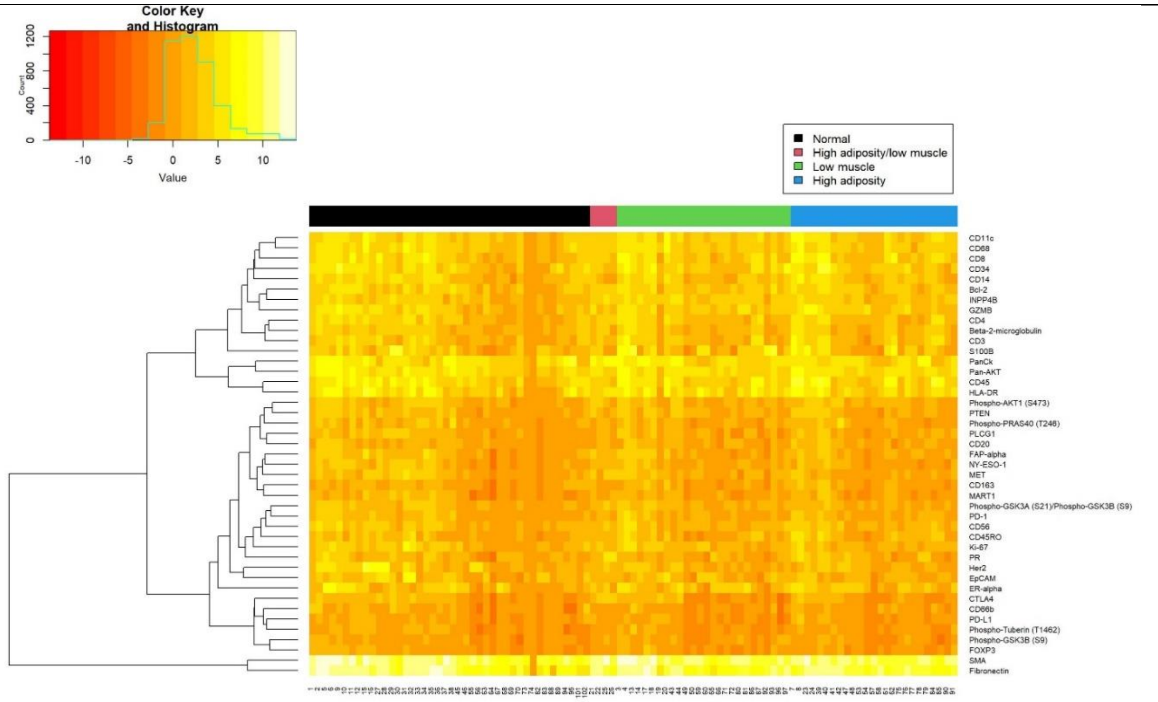

Supplemental Figure S4. Heatmap by body composition type for proteins in tumor (A) epithelium and (B) stroma

A

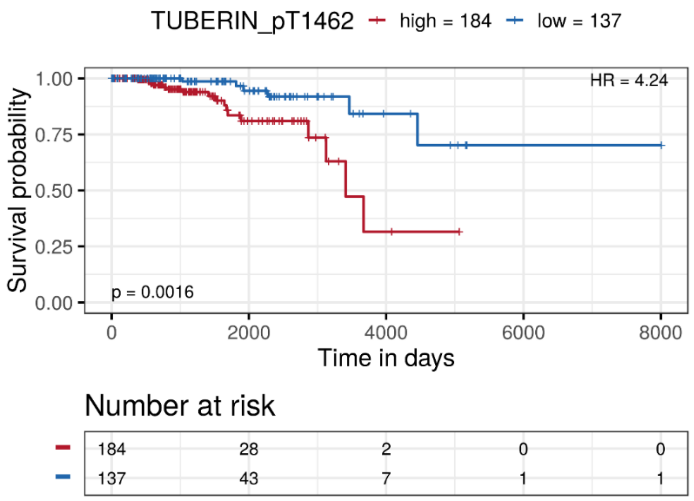

TUBERIN_pT1462 high = 184 low = 137
HR = 4.24
p = 0.0016
Survival probability
1.00
0.75
0.50
0.25
0.00
0
2000
4000
6000
8000
Time in days
Number at risk
184 28 2 0 0
137 43 7 1 1

B

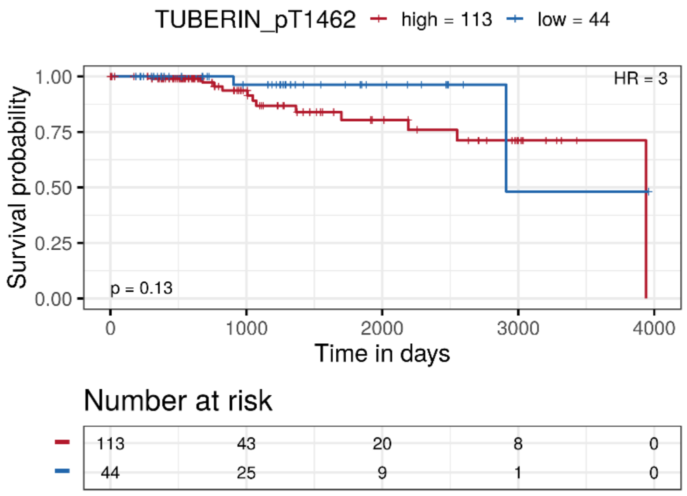

TUBERIN_pT1462 high = 113 low = 44
HR = 3
p = 0.13
Survival probability
1.00
0.75
0.50
0.25
0.00
0
1000
2000
3000
4000
Time in days
Number at risk
113 43 20 8 0
44 25 9 1 0

C

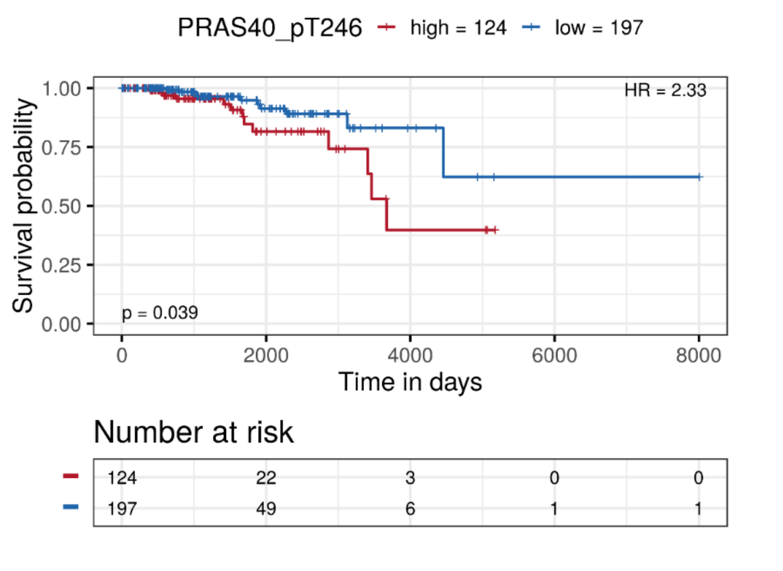

D

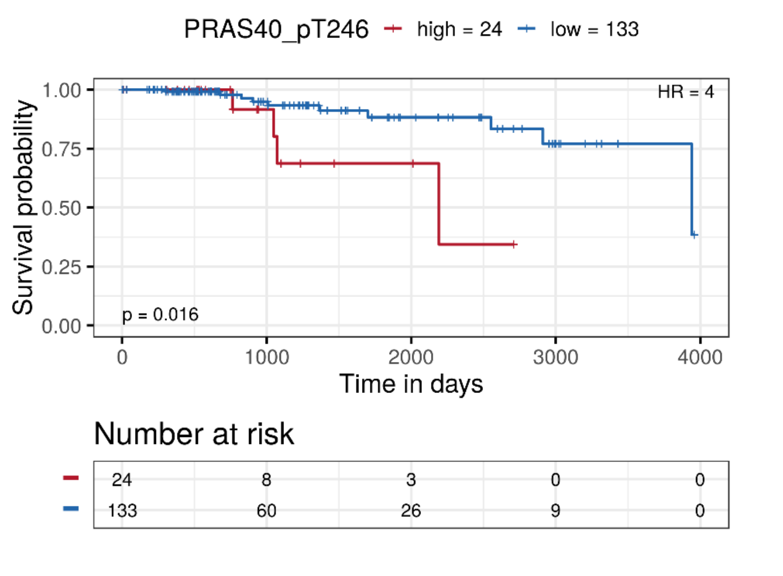

Supplemental Figure S5. Survival curves for (A) phospho-tuberin T1462 in luminal A breast cancer, (B) phospho-tuberin T1462 in luminal B breast cancer, (C) phospho-PRAS40 T246 in Luminal A breast cancer, and (D) phospho-PRAS40 T246 in Luminal B breast cancer. End points are breast cancer-specific survival. High and low levels of the proteins are derived by the optimal cutoff method. Source: The Cancer Protein Atlas: https://nborcherding.shinyapps.io/TRGAted/.

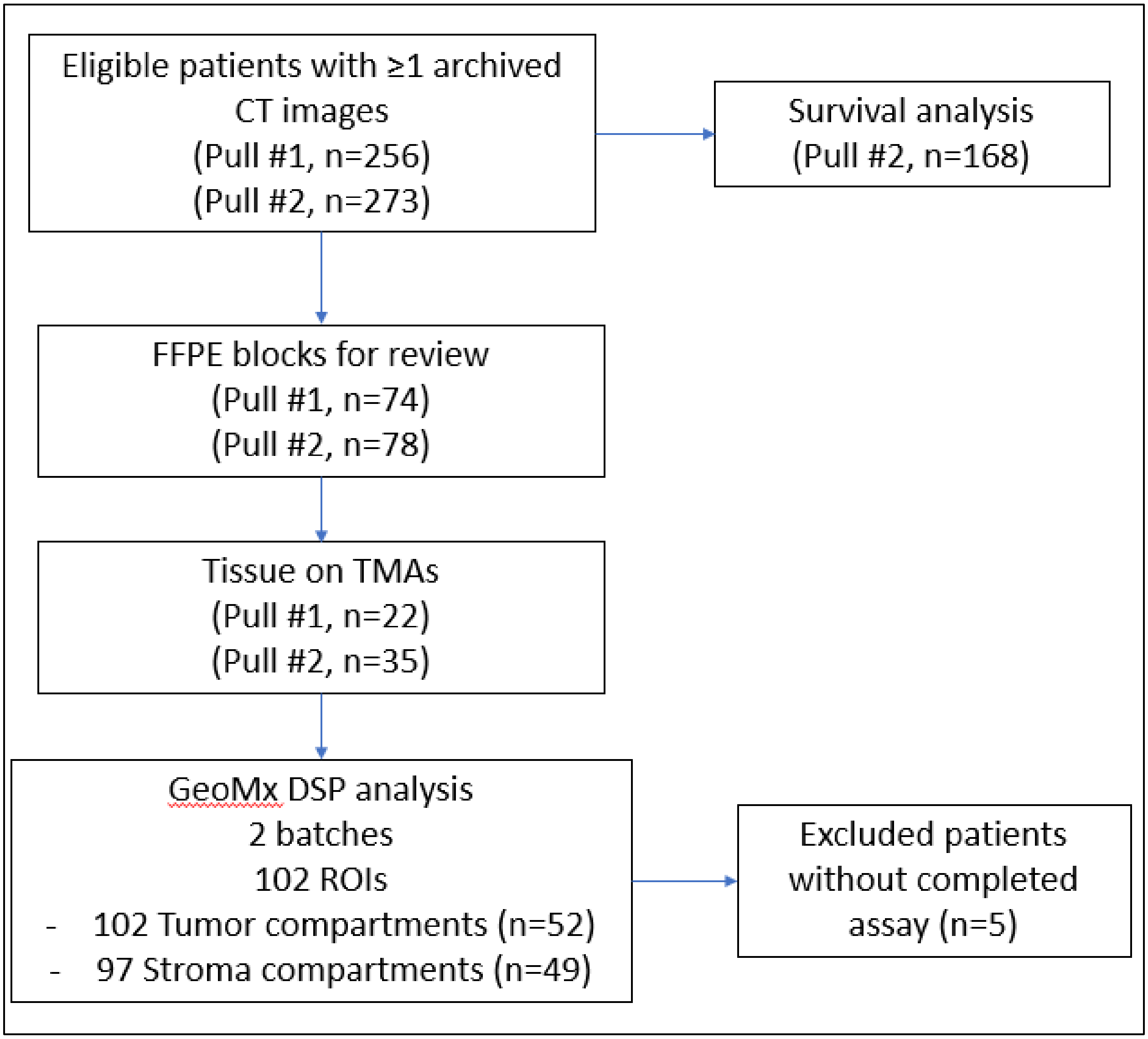

Supplemental Figure S6. Flowchart of study participants and tumor tissue sample selection

Abbreviations: CT, computed tomography; DSP, Digital Spatial Profiler; FFPE, formalin-fixed, paraffin-embedded; ROIs, regions of interest; TMA, tissue microarray.
